# Optimization of extraction and nanoencapsulation of kimchi cabbage by-products to enhance the simulated *in vitro* digestion of glucosinolates

**DOI:** 10.1016/j.heliyon.2023.e16525

**Published:** 2023-05-30

**Authors:** Sung Jin Park, Min Jung Lee, Yun-Jeong Choi, Ye-Rang Yun, Mi-Ai Lee, Sung Gi Min, Hye-Young Seo, Dong Hyeon Park, Sung Hee Park

**Affiliations:** World Institute of Kimchi, Gwangju 61755, Republic of Korea

**Keywords:** Kimchi cabbage by-products, Glucosinolates, Chitosan-lipid nanoparticles, Simulated *in vitro* digestion, Release

## Abstract

Kimchi cabbage is a well-known glucosinolate (GLS)-containing vegetable, but its by-products are discarded despite the presence of GLS. The aim of this study was the optimization of the extraction and nanoencapsulation of GLS from kimchi cabbage by-products to enhance the intestinal absorption of GLS. The optimal GLS extraction conditions included steaming thrice as pretreatment, utilizing 70% methanol, and ultrasonication at 20% amplitude for 15 min. Under these conditions, 80.11 ± 4.40 mg/100 g of GLS extraction was obtained and the extraction yield was 81.70 ± 4.73%. The optimized kimchi cabbage by-product extract (KCE) was coated with chitosan-lipid nanoparticles (KCE-NPs) and their stability and release under simulated *in vitro* gastrointestinal conditions were evaluated. KCE-NPs protected the encapsulated GLS under acidic gastric conditions and released 91.63 ± 0.76% of GLS in the simulated intestinal medium. Therefore, the proposed KCE-NPs are a promising delivery system for increasing GLS absorption.

## Highlights

• Optimization of extraction and nanoencapsulation of GLS was studied.
• Kimchi cabbage by-product extract (KCE) was investigated.
• Release profile of KCE coated with chitosan-lipid NPs (KCE-NPs) was evaluated.
• KCE-NPs protected the encapsulated GLS under acidic gastric conditions.
• KCE-NPs released 91.63 ± 0.76% of GLS in simulated intestinal medium.

## 1 Introduction

Vegetable wastes have received growing attention in the last few years owing to the possibility of extracting bioactive compounds [1]. Polyphenols are constituents of the human diet, and vegetables are the major dietary sources of these bioactive compounds. Many health benefits of vegetables consumption are thought to be due to their antioxidant properties [2]. It has been reported that fruit and vegetable wastes and by-products are abundant in bioactive compounds [[3], [4], [5]].

Cruciferous vegetables, including cabbages, have been reported the different cultivated types of cabbages also possesses significant amounts of antioxidants such as ascorbic acid, phenolic compounds and tocopherols [6,7]. Kimchi cabbage is the most popular cruciferous vegetable and two million tons of Kimchi cabbage are consumed annually in South Korea. It is prepared by salted cabbage with various seasonings and additives such as red pepper powder, garlic, ginger, green onion, fermented seafood, and glutinous rice paste [8]. However, the outer leaves and core of cabbages are discarded and treated as wastes in Kimchi manufacturing. In many cases, up to 40% of the outer leaves and core of Kimchi cabbages are discarded although they contain health-promoting phytochemicals [9]. Kimchi cabbage by-products are easily contaminated because of high water content and are associated with economic loss and environmental problems [10]. Moreover, most of the kimchi cabbage by-products are discarded despite the presence of glucosinolates (GLS). Only a few studies on the utilization of kimchi cabbage by-products as feed for animals and food fiber have been conducted [11].

GLS are sulfur-rich secondary plant metabolites in *Brassica* vegetables, e.g., cabbage, broccoli, cauliflower, radish, mustard, turnip, and rutabaga, which show a protective effect against cancer, particularly bladder, colon, and lung cancer [12,13]. Several previous studies have reported that the consumption of *Brassica* vegetables is associated with a reduction of cancer risk [14,15]. GLS are a large group of plant secondary metabolites with nutritional effects, and are mainly found in cruciferous plants. Kimchi cabbage is the most popular *Brassica* vegetable and is a well-known as GLS-containing vegetable [16,17].

It should be noted that the GLS compounds are unstable under various environments [18,19]. In particular, ingested GLS is affected by the pH and enzymes of the digestive system in the body. In some animal experiments, ingested GLS has been reported to degrade in the stomach and is partially absorbed in the intestine [[20], [21], [22]]. Nanoencapsulation is desirable to increase the bioavailability and stability of drugs in a simulated *in vitro* digestion system [23,24]. Polysaccharides and lipids are representative wall materials for nanoencapsulation, and these are used individually or in combination, depending on the characteristics and functions of the core materials [25]. Numerous nanoencapsulation strategies have been investigated, including the production of different nanocarriers such as nanoemulsions, nanostructured lipid carriers, solid lipid nanoparticles, and biopolymers [26]. Among the biopolymers, chitosan has received considerable attention owing to its nontoxic, biocompatible, biodegradable, and mucoadhesion [27]. Particularly, the mucoadhesion of chitosan enhances the intestinal absorption of bioactive compounds [28,29]. Although many studies have reported the beneficial effects of GLS on health, GLS is unstable during digestion. Therefore, the ultimate goal of this study was to optimize the extraction of GLS from kimchi cabbage by-products and enhance the intestinal absorption of GLS by nanoencapsulation.

## 2 Materials and methods

### 2.1 Materials

Kimchi cabbage by-products (outer leaves and cortex) were collected from a kimchi manufacturing plant (Sup. Fig. 1 a, b). (−)-Sinigrin hydrate (GLS standard) and chitosan **(**low molecular weight, 50,000–190,000 Da) were purchased from Sigma-Aldrich (St. Louis, MO, USA). HPLC-grade water and methanol (MeOH) were purchased from Merck & CO., Inc. (Kenilworth, NJ, USA). Medium chain triglyceride (MCT), glycerol monostearate (GMS), and tween 80 (T80) were obtained from Daejung Chemicals (Seoul, Korea). Lipases from porcine pancreas, bile extract porcine, pepsin from porcine gastric mucosa, and pancreatin from porcine pancreas were purchased from Sigma-Aldrich (St. Louis, MO, USA).

**Fig. 1:**
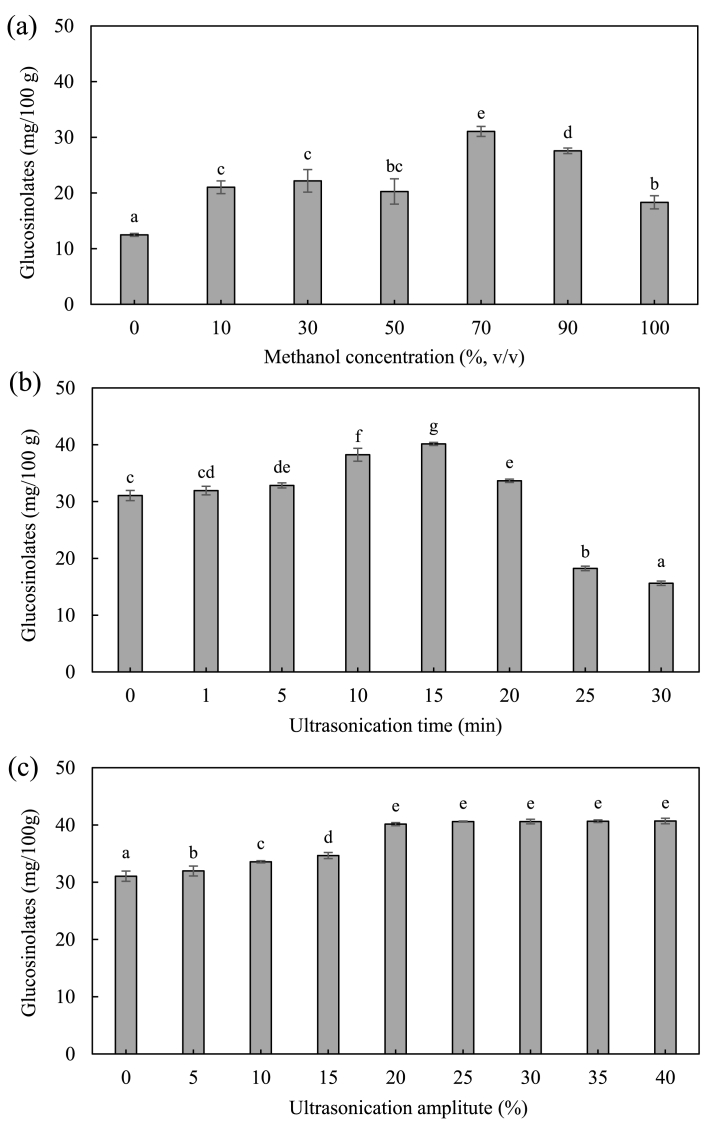
Effect of extraction conditions on GLS content: (a) methanol concentration, (b) ultrasonication, (c) time, and (c) amplitude. Different letters indicate a significant difference at *p < 0.05* (n = 3).

### 2.2 Preparation of Kimchi cabbage by-products extract (KCE)

To extract GLS from kimchi cabbage by-products, steaming was used as a pretreatment based on previous study [30]. Cotton pouches containing 100 g of kimchi cabbage by-products were prepared and steamed using an autoclave, which was set to reach a temperature of 120 °C for 11 min (Prestige classic autoclave, Duraline Systems, Inc., NY, USA). After steaming, the kimchi cabbage by-products were dried at 80 °C for 48 h. This process was repeated 1–12 times. The steamed kimchi cabbage by-products were extracted using 0–100% MeOH and ultrasonication (for 0–30 min at 0–40% amplitude). The resulting extract was centrifuged at 10,000 rpm and 4 °C for 10 min. The supernatant was collected, evaporated, and freeze-dried.

### 2.3 Determination of total GLS

Total GLS was measured using the method described by Mawlong et al. [31], with slight modifications. Briefly, the samples and (−)-sinigrin (glucosinolates standard) were mixed with 2 mM sodium tetrachloropalladate (sodium tetrachloropalladate and 170 μL of concentrated HCL, and 100 mL of double distilled water). The mixtures were incubated at 25 °C for 1 h and centrifuged at 3000 rpm for 4 min. The supernatant was collected and measured at 425 nm using a UV-spectrophotometer (Shimadzu UV-1900, Kyoto, Japan).

### 2.4 Preparation of KCE-loaded chitosan-lipid nanoparticles (KCE-NPs)

KCE-NPs were prepared by emulsification and homogenization. Briefly, an aqueous surfactant phase consisting of KCE, double distilled water, and Tween 80 (T80) was prepared and heated to 75 °C before adding it to the lipid phase. Simultaneously, glycerol monostearate (GMS) and medium chain triglyceride (MCT) were prepared and heated up to 80 °C prior to mixing with the aqueous phase. The hot aqueous phase was added to the lipid and homogenized at 80 °C for 2 h. Chitosan solution was prepared by adding 0.5 g chitosan to 100 mL of lactic acid solution and stirring for 24 h, to ensure complete solubilization. Subsequently, the mixtures were coated with chitosan solutions and homogenized by high-pressure homogenization (EmulsiFlexVR -C3, AVESTIN, Ottawa, Canada) at 10,000 psi and 70 °C for three cycles resulting in the formulation of KCE-NPs. The optimal formulation of lipids, chitosan and KCE was determined by the separation of layers in the KCE solution and the encapsulation efficiency of KCE-NPs after storage for a day at room temperature (25 °C) (Sup. Table 1).

**Table 1:** The encapsulation efficiency by formulation of KCCE-NP. Different letters indicate a significant difference at *p < 0.05 (n =3).*

Formulation					Encapsulation efficiency (%)
MCT (%)	GMS (%)	T80 (%)	CS (%)	KCE (%)	
80	0	20	0	10	−^1)^
72	8	20	0	10	–
64	16	20	0	10	–
56	24	20	0	10	–
48	32	20	0	10	–
40	40	20	0	10	35.44 ± 1.22^a^
32	48	20	0	10	47.77 ± 2.11^b^
24	56	20	0	10	40.85 ± 3.57^ab^
16	64	20	0	10	37.75 ± 2.63^a^
8	72	20	0	10	–
0	80	20	0	10	–
32	48	20	5	10	47.68 ± 2.29^b^
32	48	20	10	10	56.86 ± 5.66^c^
32	48	20	15	10	89.31 ± 2.15^d^
32	48	20	20	10	84.00 ± 6.04^d^
32	48	20	25	10	63.25 ± 5.42^c^

^1)^ Hyphen (−) was means the ingredients are separated rather than mixed.

### 2.5 Encapsulation efficiency of KCE-NPs

The encapsulation efficiency of the KCE-NPs was determined as described earlier with slight modifications [32]. The formulations of the KCE-NPs are presented in Table 1. Briefly, the fabricated KCE-NPs (1 mL) were added to 9 mL of 70% MeOH and centrifuged at 10,000 rpm for 10 min, and the supernatant was collected for quantitative analysis of non-encapsulated core material (free GLS). The obtained supernatants were analyzed using a UV-spectrophotometer (425 nm). Encapsulation efficiency (EE) was calculated using the following equation: Encapsulation efficiency (%) = (Total GLS-Free GLS)/Total GLS × 100.

### 2.6 Stability of KCE-NPs under simulated *in vitro* digestion

The KCE-NPs were applied to the simulated *in vitro* digestion medium to investigate the influence of the chitosan-lipid coating on the digestion stability. First, the simulated gastric and intestinal mediums (SGM and SIM, respectively) were prepared, according to a previous report [33], with slight modifications. Briefly, SGM was prepared by mixing 0.32% (w/v) pepsin, 2 g sodium chloride and 7 mL HCL in 1 L distilled water and adjusting to pH 2 using 1 M HCL. SIM was prepared by mixing 0.4 mg/mL lipase, 0.7 mg/mL bile extract solution, 0.5 mg/mL pancreatin, and 1 mL?50 mM calcium chloride solution, and adjusting the pH 7 ± 0.1 using 0.1 M NaOH. The KCE-NPs (5 mL) were mixed with 5 mL of SGM at 37 °C for 2 h, followed by mixing 10 mL of SIM at 37 °C for 2 h. Each mixture was evaluated for stability by analyzing the particle diameter, polydispersity index (PDI), and zeta potential. The mean particle diameter, PDI, and zeta potential were measured by dynamic light scattering using the zeta-potential and particle size analyzer (ELSZ-1000, Otsuka Electronics Co., Ltd., Osaka, Japan) at a fixed detector angle of 90° and at 25 °C. Each sample was measured at thrice, and the average values were used.

### 2.7 In vitro release

Dialysis sacks (MWCO 12,000 Da, Sigma-Aldrich) were used to evaluate the release of GLS from the KCE and KCE-NPs as described in a previous report [34]. The dialysis sacks were soaked in distilled water overnight, and then filled with the samples and digestive media. Both ends of the sacks were tied to prevent any leakage, and the sacks were carefully placed in a beaker containing 30 mL of methanol and incubated at 37 °C for 0, 2, 4, 6, 8 and 10 h while stirring at 50 rpm. The samples were first incubated in SGM and then re-suspended in SIM. A 1-mL aliquot was taken at each time interval and replaced with fresh medium. The amount of GLS released from the KCE and KCE-NPs was compared.

### 2.8 Morphology of KCE-NPs under *in vitro* digestion

The morphology of the KCE-NPs was determined by transmission electron microscopy (TEM; HT 7700, Hitachi, Tokyo, Japan). Imaging was performed at 100 kV and magnification of × 80.0 K. A drop of sample was placed on the carbon-coated grids (Ted Pella, Redding, CA, USA) and the excess was removed with filter paper. This procedure was repeated thrice. Finally, the grids were stained with 1% (w/v) phosphotungstate for 1 min and dried at 25 °C for 12 h.

### 2.9 Statistical analysis

All results were obtained in triplicate and the data were expressed as mean ± standard deviation. Statistical analysis was performed using Statistical Package for the Social Science (SPSS, Version 20.0, SPSS Inc.,Chicago, IL, USA). Analysis of variance (ANOVA) and Duncan's multiple range tests were used to determine the statistical significance among the means at 95% significant level.

## 3 Results and discussion

### 3.1 Preparation of Kimchi cabbage by-products extract (KCE)

**Fig. 2:**
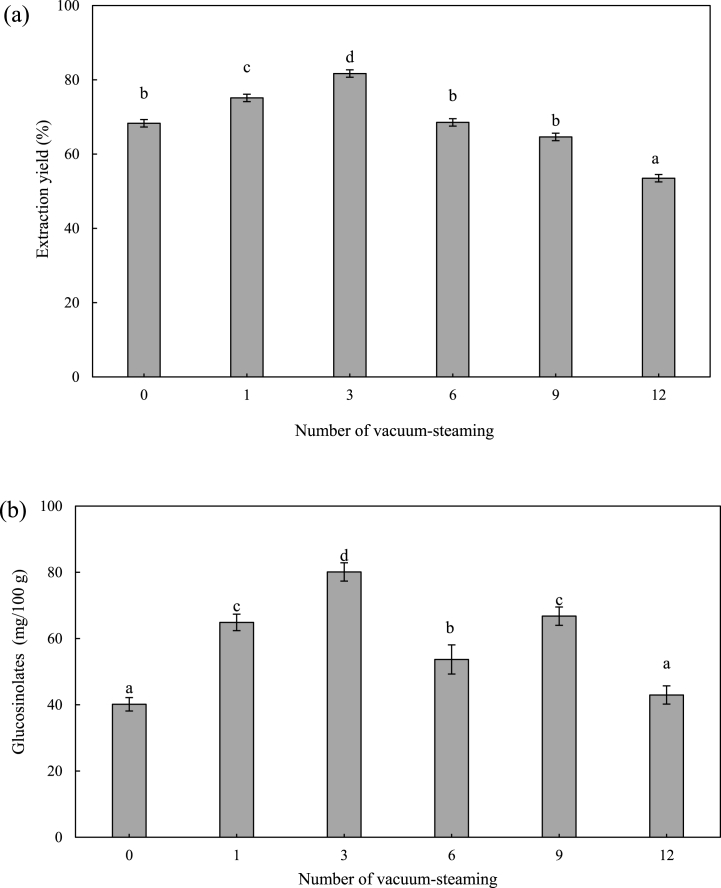
Effect of steaming treatment on (a) extraction yield (%) and (b) GLS. Different letters indicate a significant difference at *p < 0.05 (n =3).*

The purpose of this study was to use the waste kimchi cabbage by-products as sources of GLS. Therefore, it is necessary to prepare extracts with high concentrations of GLS. The pretreatment, extraction solvent, and processing methods were optimized to extract GLS from the kimchi cabbage by-products. In this study, steaming as pretreatment, 70% MeOH as solvent, and ultrasonication were used. The highest content of GLS (31.05 ± 0.90 mg/100 g) was extracted using 70% MeOH extraction and the content of GLS decreased when >70% MeOH was used (Fig. 1a). Physical extraction using ultrasonication further increased the GLS content compared to KCE without treatment. The optimal ultrasonication time (min) and power (amplitude) were found to be 15 min and 20% amplitude, respectively (Fig. 1 b, c). Therefore, the optimal extraction conditions involved the use of 70% MeOH with ultrasonication at 20% amplitude for 15 min (40.60 ± 0.27 mg/100 g). Several studies have reported that heat treatment by steaming was increases GLS extraction in *Brassica* vegetables [19]. Thus, the number of steaming cycles were optimized by evaluating the GLS content and extraction yield. The highest extraction yield (81.70 ± 4.11%) and GLS amounts (80.11 ± 4.40 mg/100 g) are achieved by steaming three-times (Fig. 2a and b). Consequently, 70% MeOH and ultrasonication at 20% amplitude for 15 min is were found to be the most optimal conditions for KCE extraction. Previous studies have reported that the use of aqueous solution of MeOH, ultrasonication, and steaming effective for the extraction of nutraceutical compounds from natural products [[35], [36], [37]]. Thus, the mixing of the methods is suitable to prepare the KCE with high GLS.

### 3.2 Optimization of the KCE-NPs

KCE had 80.11 ± 4.40 mg/100 g GLS, and GLS is unstable under acidic and gastric conditions [21,22]. Therefore, nanoencapsulation of KCE was required to increase the stability of GLS under these conditions. Chitosan and lipid mixtures (solid lipid, liquid lipid, and emulsifier) were used as wall materials for the encapsulation of KCE in this study. The formulation of KCE-NPs was optimized by the separation of layers of KCE-NPs and encapsulation efficiency of GLS (Table 1). First, the mixing ratio of medium chain triglyceride (MCT) and glycerol monostearate (GMS) was optimized. At a mixing ratio of 2:3 (MCT: GMS), no layer separation was observed and the highest encapsulation efficiency (47.77 ± 2.11%) in various lipid mixtures was obtained. These results are consistent with data in a previous study [37], but the encapsulation efficiency is very low (>50%). However, the encapsulation efficiency increases significantly with chitosan coating; the addition of 15 mg of chitosan in optimum lipid mixtures resulted in the highest encapsulation efficiency (89.31 ± 2.15%). Consequently, the optimized KCE-NP formulation included MCT (32 mg), GMS (48 mg), Tween 80 (20 mg) and chitosan (15 mg), and after high-pressure homogenization, the mean particle diameter, PDI, and zeta potential of KCE-NPs were 205.58 ± 9.08 nm, 0.25 ± 0.013, and 32.63 ± 0.45 mV, respectively. The mean particle diameter, size distribution and zeta potential are important parameters for the stability, release, and bioavailability of GLS. Generally a nanoparticle diameter of ≤ 200 nm, PDI of <0.3, and zeta potential of > ±30 mV is preferred for drug delivery because of their high residence time in the gastrointestinal tract and increased solubility and reactivity [38] Thus, the prepared KCE-NPs were homogeneous.

### 3.3 Stability of KCE-NPs in simulated *in vitro* gastrointestinal medium

**Fig. 3:**
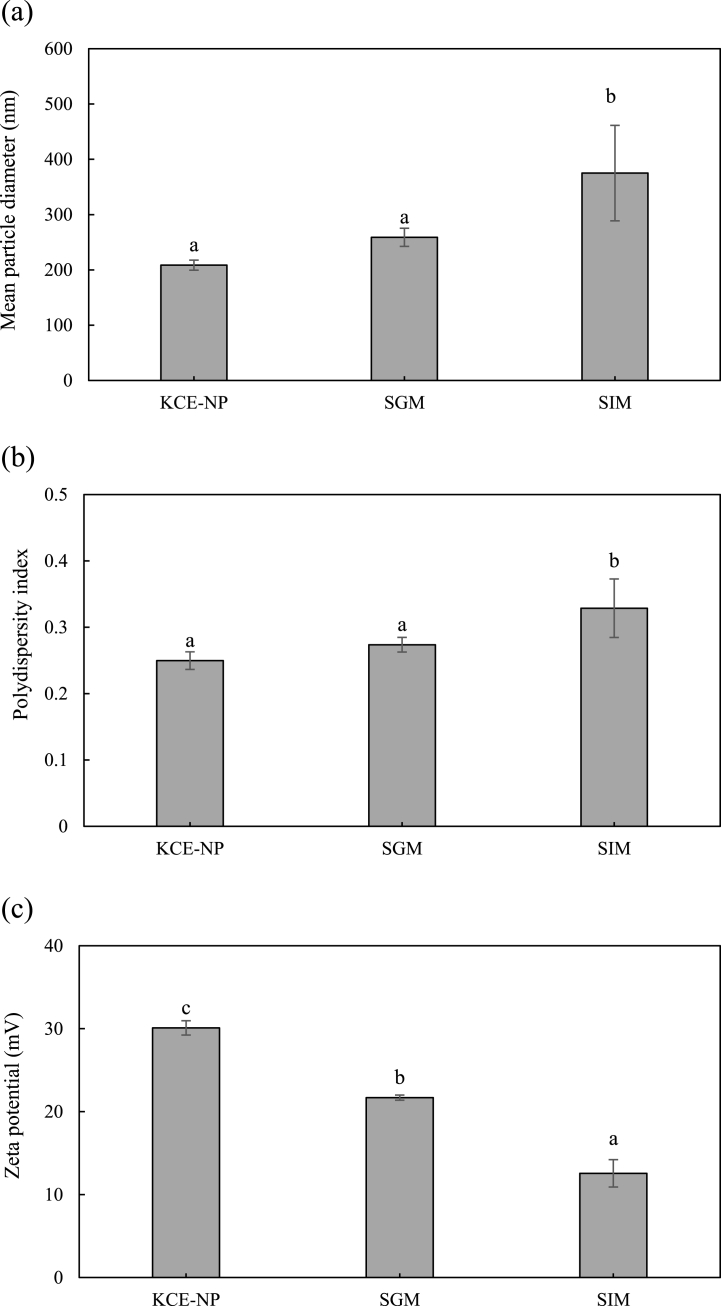
Stability of KCE-NPs in simulated *in vitro* digestion (simulated gastric medium (SGM) and simulated intestinal medium (SIM)). (a) Mean particle diameter, (b) polydispersity index, and (c) zeta potential. Different letters indicate a significant difference at *p < 0.05* (n = 3).

**Fig. 4:**
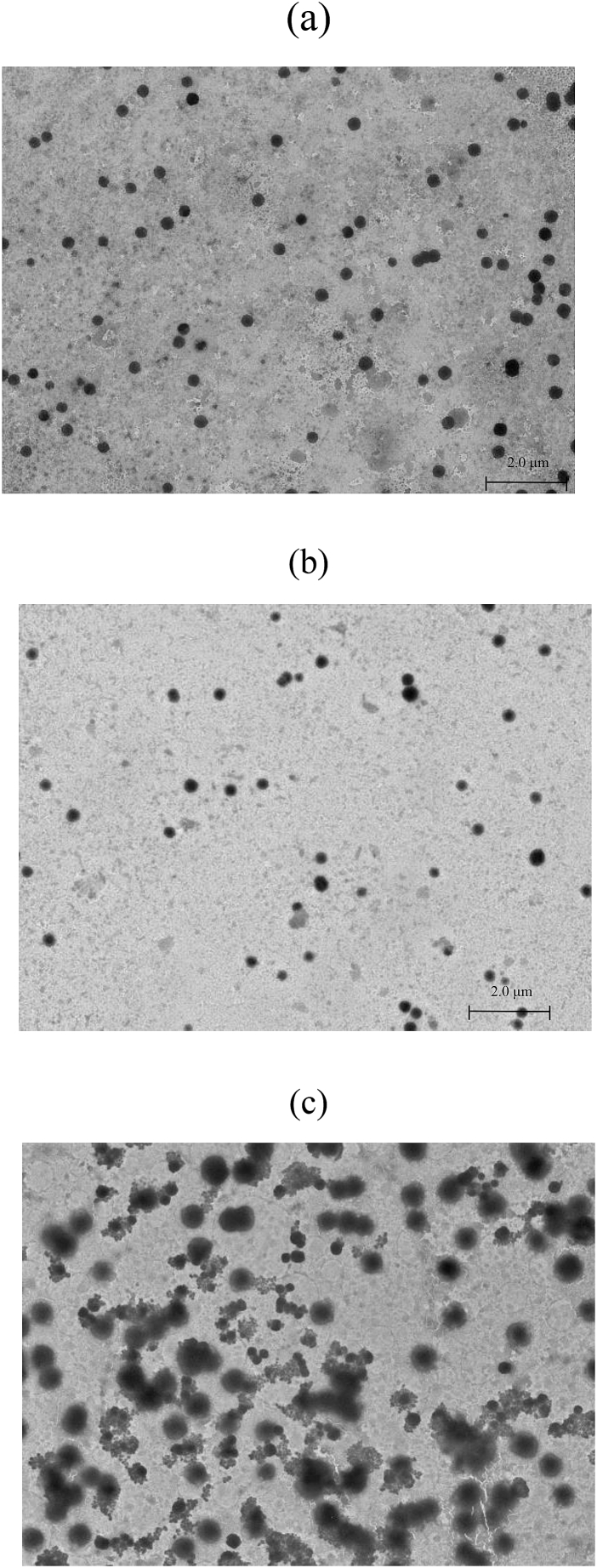
Changes in the morphology of KCE-NPs after digestion in simulated gastric medium (SGM; pH 2.0, 37 °C, 2 h) and simulated intestinal medium (SIM; pH 7.0, 37 °C, 2 h).

The stability of KCE-NPs in simulated *in vitro* gastrointestinal medium was evaluated by measuring the mean particle diameter, zeta potential, PDI, and morphology of KCE-NPs in SGM and SIM. As the KCE-NPs passed through SGM and SIM, the mean particle diameter and PDI increased, whereas the zeta potential decreased. These results are due to the presence of pepsin and bile salts, which have negatively charged molecules on their surfaces. Passage of KCE-NPs through SGM and SIM, resulted in the attachment of enzymes and bile salts to KCE-NPs (Fig. 3 a-c). The changes in KCE-NPs in these media were observed by TEM. The KCE-NPs had a mean particle diameter of about 200 nm and spherical morphology in initial and SGM. However, after interaction with SIM, aggregation of KCE-NPs was observed (Fig. 4a–c). These results indicate electrostatic interactions between the positive charge of chitosan molecule in KCE-NPs and negative charges of the digestive enzymes in SIM [39]. Thus, KCE-NPs can protect GLS in SGM and form aggregates due to interactions with the digestive enzymes in SIM, allowing the controlled release of GLS.

### 3.4 Release of GLS

**Fig. 5:**
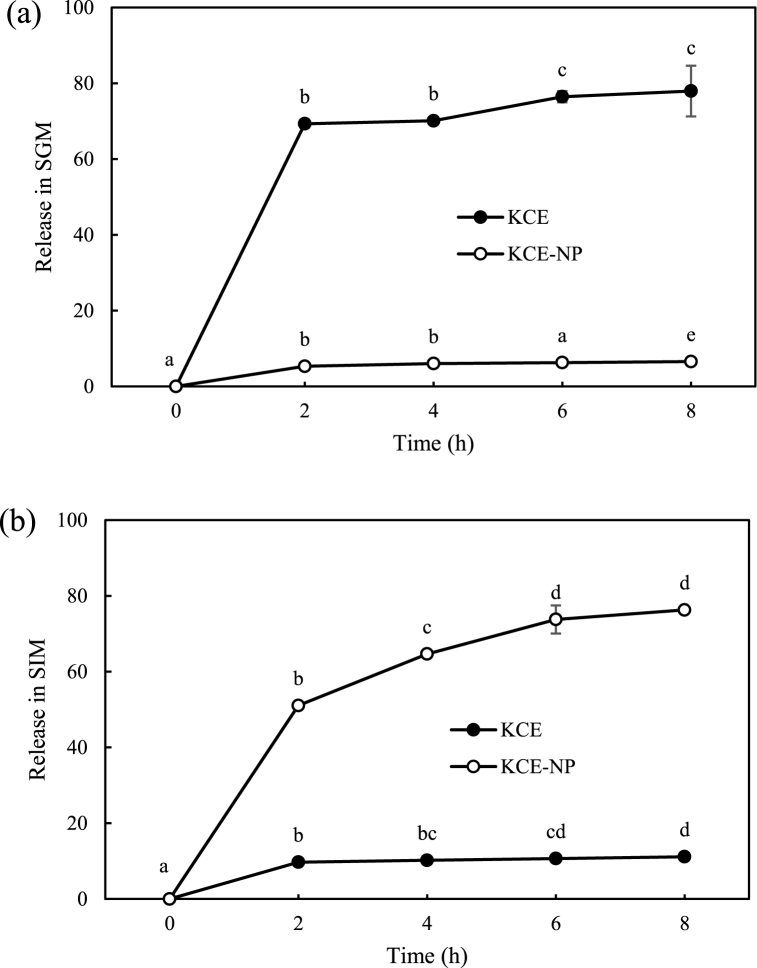
*In vitro* release profiles of GLS from KCE-NPs and KCE. (a) SGM and (b) SIM. Different letters indicate a significant difference at *p < 0.05* (n = 3).

The sustained release of GLS from KCE-NPs in SGM and SIM over a period of 8 h was compared with that from KCE (Fig. 5a and b). In SGM, 6.55 ± 0.07% and 77.95 ± 6.71% of the GLS was released from the KCE-NPs and KCE, respectively. In SIM, 91.63 ± 0.76% of GLS was released from the KCE-NPs, whereas only 11.13 ± 0.56% of GLS was released from KCE. The release profile of KCE-NPs shows that GLS was effectively released in SIM. Hence, KCE-NPs can be considered as effective GLS carriers because they prevented the degradation of GLS in SGM and increased the bioavailability of GLS. GLS are broken down by the enzymes in the colon and the metabolites are absorbed in the small intestine [38]. The main role of KCE-NPs in these systems is the regulation of GLS release kinetics to achieve a sustained and controlled release. There are several kinetic models including zero to fifth order polynomials, Korsmeyer-Peppas, Weibull and hyperbolic tangent function that can be used to explain the drug release o from polymer based nanoparticles [39,40]. Among them, the hyperbolic tangent function model as the most adequate and general model to describe the GLS release kinetics [41]. Therefore, the enhancement of GLS release in SIM is related to the bioavailability of GLS. Shin and Kim [29] reported the effects of chitosan coating on the bioavailability of curcumin in simulated *in vitro* digestion systems. Additionally, Park et al. [42] reported the successful coating of turmeric powder by solid and liquid lipid mixture carriers, and the improvement of the bioavailability of curcumin in simulated *in vitro* digestion systems. Hence, the KCE-NPs can increase the bioavailability of GLS under simulated *in vitro* digestion systems.

## 4 Conclusions

In this study, KCE was successfully obtained from the kimchi cabbage by-products and KCE-NPs were fabricated. GLS extraction from treated KCE was significantly higher (40.60 ± 0.09 mg/100 g) than that from KCE without treatment (12.48 ± 0.26 mg/100 g). The digestion of KCE-NPs during simulated *in vitro* digestion demonstrates their controlled release capability as KCE-NPs can protect the GLS in SGM; however, we observed release of more than 90% GLS from KCE-NPs after 8 h of digestion in SIM. Therefore, the developed KCE-NPs can be effectively applied for encapsulating and increasing the bioavailability of GLS. Furthermore, the results show that the kimchi cabbage by-products are a good source of GLS. The accumulation of vegetable wastes and by-products has become a serious global problem causing as environmental pollution, processing cost issues, and money, and others. Although, the use of kimchi cabbage by-product was presented in this study, further studies on economic feasibility and mass production will be required. Furthermore, additional verification experiments are needed for the efficacy of encapsulated GLS and its bioavailability in real animal models.

## Funding statement

This is research was supported by a grant from the World Institute of Kimchi (KE2302-1 & KE 2302-3), funded by the Ministry of Science, ICT, Republic of Korea.

## Declaration

### Author contribution statement

Sung Jin Park, Mi-Ai Lee: Conceived and designed the experiments; Wrote the paper.

Min Jung Lee, Yun-Jeong Choi: Performed the experiments.

Ye-Rang Yun, Dong Hyeon Park: Analyzed and interpreted the data.

Sung Gi Min, Hye-Young Seo: Contributed reagents, materials, analysis tools or data.

Sung Hee Park: Conceived and designed the experiments.

### Data availability statement

Data included in article/supplementary material/referenced in article.

## Declaration of competing interest

The authors declare that they have no known competing financial interests or personal relationships that could have appeared to influence the work reported in this paper.

## Appendix A Supplementary data

Sup. Figure. 1 Kimchi cabbage by-products. (a) outer leaves and (b) core. Sup. Figure. 1
