# Supplementary figures and images for: Optimization of extraction and nanoencapsulation of kimchi cabbage by-products to enhance the simulated in vitro digestion of glucosinolates
Source: Heliyon. 2023 May 30;9(6):e16525. doi: 10.1016/j.heliyon.2023.e16525 (PMC10360592; doi:10.1016/j.heliyon.2023.e16525)

(a)

**
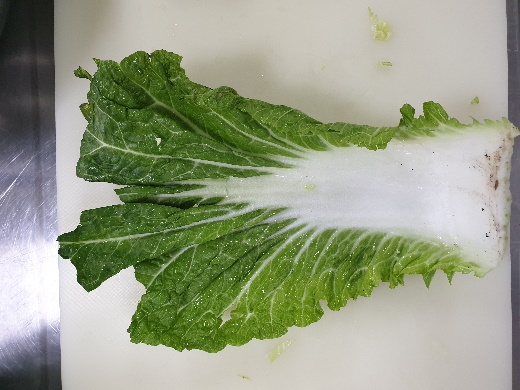
**

(b)


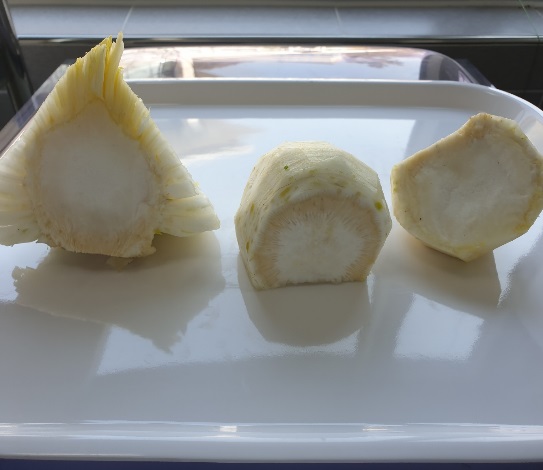


**Supplementary Figure 1.**

Supplement: Sup. Figure. 1 — Kimchi cabbage by-products. (a) outer leaves and (b) core. [file mmc1.docx]
